# Association between interstitial lung abnormality and mortality in patients with esophageal cancer

**DOI:** 10.1007/s11604-024-01563-x

**Published:** 2024-04-25

**Authors:** Akinori Hata, Masahiro Yanagawa, Tomo Miyata, Yu Hiraoka, Motohiro Shirae, Keisuke Ninomiya, Shuhei Doi, Kazuki Yamagata, Yuriko Yoshida, Noriko Kikuchi, Ryo Ogawa, Hiroto Hatabu, Noriyuki Tomiyama

**Affiliations:** 1 Department of Diagnostic and Interventional Radiology, Graduate School of Medicine, Osaka University, 2-2 Yamadaoka, Suita, Osaka 5650871 Japan; 2 Department of Radiology, Sakai City Medical Center, 1-1-1 Ebaraji-cho, Nishi-ku, Sakai, Osaka 5938304 Japan; 3 Future Diagnostic Radiology, Graduate School of Medicine, Osaka University, 2-2 Yamadaoka, Suita, Osaka 5650871 Japan; 4 Center for Pulmonary Functional Imaging, Department of Radiology, Brigham and Women’s Hospital and Harvard Medical School, 75 Francis Street, Boston, MA 02115 USA

**Keywords:** Chest, Esophageal cancer, Computed tomography, X-ray, Interstitial lung disease, Interstitial lung abnormalities

## Abstract

**Purpose:**

To investigate the relationship between interstitial lung abnormalities (ILAs) and mortality in patients with esophageal cancer and the cause of mortality.

**Materials and methods:**

This retrospective study investigated patients with esophageal cancer from January 2011 to December 2015. ILAs were visually scored on baseline CT using a 3-point scale (0 = non-ILA, 1 = indeterminate for ILA, and 2 = ILA). ILAs were classified into subcategories of non-subpleural, subpleural non-fibrotic, and subpleural fibrotic. Five-year overall survival (OS) was compared between patients with and without ILAs using the multivariable Cox proportional hazards model. Subgroup analyses were performed based on cancer stage and ILA subcategories. The prevalences of treatment complications and death due to esophageal cancer and pneumonia/respiratory failure were analyzed using Fisher’s exact test.

**Results:**

A total of 478 patients with esophageal cancer (age, 66.8 years ± 8.6 [standard deviation]; 64 women) were evaluated in this study. Among them, 267 patients showed no ILAs, 125 patients were indeterminate for ILAs, and 86 patients showed ILAs. ILAs were a significant factor for shorter OS (hazard ratio [HR] = 1.68, 95% confidence interval [CI] 1.10–2.55, P = 0.016) in the multivariable Cox proportional hazards model adjusting for age, sex, smoking history, clinical stage, and histology. On subgroup analysis using patients with clinical stage IVB, the presence of ILAs was a significant factor (HR = 3.78, 95% CI 1.67–8.54, P = 0.001). Subpleural fibrotic ILAs were significantly associated with shorter OS (HR = 2.22, 95% CI 1.25–3.93, P = 0.006). There was no significant difference in treatment complications. Patients with ILAs showed a higher prevalence of death due to pneumonia/respiratory failure than those without ILAs (non-ILA, 2/95 [2%]; ILA, 5/39 [13%]; P = 0.022). The prevalence of death due to esophageal cancer was similar in patients with and without ILA (non-ILA, 82/95 [86%]; ILA 32/39 [82%]; P = 0.596).

**Conclusion:**

ILAs were significantly associated with shorter survival in patients with esophageal cancer.

**Supplementary Information:**

The online version contains supplementary material available at 10.1007/s11604-024-01563-x.

## Introduction

Interstitial lung abnormalities (ILAs) are defined as incidental radiologic patterns in the lungs on computed tomography (CT) including subtle interstitial findings [1]. ILAs do not necessarily represent a distinct disease, but many previous studies have shown the potential clinical significance of ILAs; ILAs often show imaging progression and are associated with increased respiratory symptoms, reductions of lung volume, exercise capacity, and gas exchange, and a greater risk of all-cause mortality [1–9]. Interstitial lung diseases are often irreversible [10, 11] and ILAs are considered as early or mild forms of ILD. In addition, many studies have investigated the association between ILAs and lung cancer with respect to cancer incidence [12–14], survival [12, 14–17], and treatment complications [18–23].

Esophageal cancer is the 8th most common cancer worldwide and one of the most aggressive gastrointestinal cancers [24–30]. The primary histological type of esophageal carcinoma in Asia is squamous cell carcinoma (SCC), and tobacco smoking is one of the major risk factors for development of esophageal cancer, and especially of SCC. It has been reported that smoking is also a risk factor for the presence of ILAs [4, 6, 7, 14]. In addition, patients with esophageal cancer receive a multidisciplinary approach to treatment, including surgery, chemotherapy, and radiation therapy, and these treatment options can cause complications [31]. Therefore, we hypothesized that patients with esophageal cancer are likely to present with ILAs, and ILAs may affect the clinical course of esophageal cancer.

Tseng et al. [32] investigated ILAs in patients with locally advanced esophageal cancer (N = 208), and they reported that there was no significant association between ILAs and mortality. However, most patients in their cohort were White and had adenocarcinoma. In addition, there has been no study that investigated esophageal cancer patients considering clinical stage and included patients with metastatic stage. The purpose of this study was to investigate the association between ILAs and mortality in patients with esophageal cancer including advanced stage. In addition, cause of mortality and factors that affected the difference in the prognosis between patients with and without ILAs were investigated.

## Patients and methods

### Patients

This study was approved by the institutional review board of our hospital (Osaka University Hospital) and written informed consent was waived due to its retrospective nature. All methods were carried out in accordance with relevant guidelines and regulations.

**Fig. 1:**
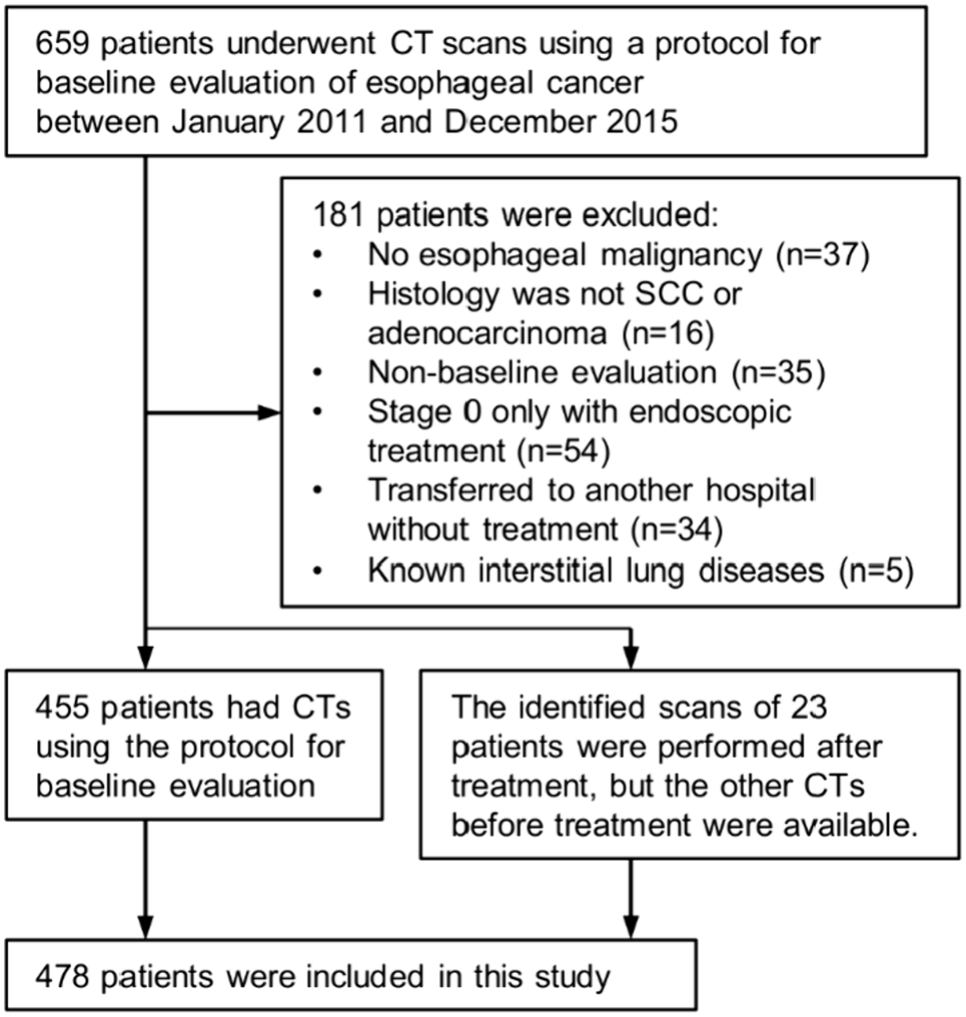
Flowchart of patient selection

We checked consecutive patients from January 2011 to December 2015 in our picture archiving and communication system. The inclusion criterion was patients who underwent CT scans following a protocol for baseline assessment of esophageal tumors. The exclusion criteria were as follows: no esophageal malignancy; histology was not SCC or adenocarcinoma; the identified CT was performed after treatment and CT before treatment was not available; the patient transferred to another hospital without treatment in our hospital and survival data were not available; stage 0 only with endoscopic treatment; and patients with known interstitial lung diseases at the time of esophageal cancer diagnosis. A total of 478 patients were included in this study. Patient selection is summarized in Fig. 1 and the supplementary document.

### CT image acquisition and evaluation for ILA

The majority of patients (455/478) underwent CT scan using the protocol for baseline evaluation of esophageal cancer and thin slice images (0.5- or 0.625-mm thickness) were obtained. The other patients and detailed image reconstruction settings are described in the supplementary document.

**Fig. 2:**
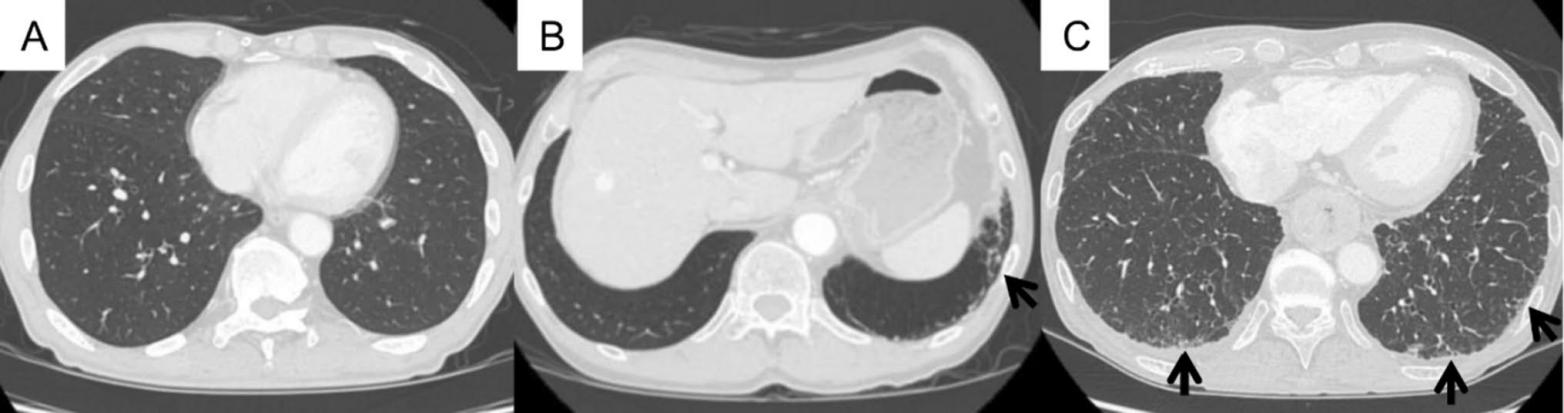
Examples of ILAs. **A** Non-ILA. A 63-year-old man. CT shows no interstitial lung findings. **B** Indeterminate for ILAs. A 71-year-old man. CT shows subtle reticular abnormality (arrow), but the lesion area is limited. **C** ILA. A 64-year-old man. CT shows ground-glass and irregular linear abnormalities in the subpleural areas of the bilateral lungs (arrows). *ILA* interstitial lung abnormality

Two chest radiologists (T.M. and A.H.), with 10 and 11 years of experience, respectively, independently interpreted CT images of each subject without knowledge of the patients’ status. Each CT scan was scored using a 3-point scale: 0, no ILA; 1, indeterminate for ILA; and 2, ILA (Fig. 2) [3]. ILAs were defined as radiologic patterns of increased lung density including non-dependent ground-glass or reticular abnormalities, non-emphysematous cysts, honeycombing, and traction bronchiectasis affecting more than 5% of any lung zone [1]. Aspiration pneumonia, suggested by nodularity or the tree-in-bud sign with a lobar or segmental distribution and with or without central plugging of airways, was excluded from ILA. The patients with apparent aspiration pneumonia with above typical findings were classified as non-ILA (score 0), and indeterminate patients were classified as indeterminate for ILA (score 1). The patients with interstitial findings in areas other than those of aspiration were classified as having ILAs (score 2). In the cases with a discrepancy between the two readers, a third chest radiologist with 20 years of experience (M.Y.) independently scored the cases with no knowledge of the patient’s status. When two of the three interpreters gave the same score, that score was used. Cases in which all three radiologists gave different scores were considered indeterminate (score 1).

In the patients with ILAs, subtypes were evaluated: non-subpleural, subpleural non-fibrotic, and subpleural fibrotic [1]. The same two readers, with 10 and 11 years of experience, respectively, independently interpreted the images again and assessed whether the ILAs were subpleural predominant or not. In the cases with subpleural predominance, they assessed whether the ILAs were accompanied by fibrotic findings (presence of architectural distortion with traction bronchiectasis or honeycombing). In the cases with a discrepancy between the two readers, the third reader with 20 years of experience made the final judgement regarding subpleural predominance and fibrosis.

### Causes of death and treatment complications

To evaluate the factors associated with worse mortality, the causes of death and treatment complications were investigated. In this investigation, death was considered to be due to original cancer progression, pneumonia and respiratory failure not in the end stage of the original cancer, or pulmonary complications during treatment. In the patients who underwent surgery, postoperative complications with Clavien-Dindo Classification grade II or more that occurred during hospitalization or readmission for 60 days postoperatively were identified. Pulmonary postoperative complications were counted separately (supplementary document). Furthermore, drug-related pneumonitis and radiation pneumonitis were investigated in the patients treated without surgery (supplementary document). In addition, the use of immune checkpoint inhibitor (ICI) was investigated.

### Statistical analysis

Interobserver agreement of ILA scoring between the first and second readers was assessed with a weighted kappa coefficient (κw), using the following categorization for kappa: poor (0 < κw ≤ 0.20), fair (0.20 < κw ≤ 0.40), moderate (0.40 < κ ≤ 0.60), good (0.60 < κw ≤ 0.80), and excellent (0.80 < κw ≤ 1.00) [33].

Demographic characteristics, including age, sex, body mass index [BMI], smoking status (never, former, and current), pack-years of cigarette smoking, cancer clinical stage (the eighth edition of the American Joint Committee on Cancer staging system), and surgery were collected from the electronic medical record system. These demographics were compared between the groups without and with ILA (scores 0 and 2). The group indeterminate for ILA was not included in the statistical analysis to simplify the results according to the previous studies [9, 14, 34]. Emphysema quantification on CT was obtained in the groups without and with ILA to adjust the influence of emphysema in the multivariable prognosis analysis (supplementary document). Age and BMI were expressed as mean ± standard deviation and compared using *t* test; %emphysema was expressed as median (interquartile range) and compared using Mann–Whitney *U* test; categorical variables are expressed as n (%) and were compared by Fisher’s exact test.

Five-year disease-free survival (DFS) and overall survival (OS) were evaluated using the Kaplan–Meier method. DFS was evaluated only in the patients who underwent surgery. Univariable and multivariable Cox proportional hazards model analyses were performed to estimate hazard ratios (HRs). For OS, subgroup analyses were performed in patients with stage I–III, those with stage IVA, and those with stage IVB. Furthermore, additional analysis of subcategories (non-ILA vs. non-subpleural and subpleural non-fibrotic ILAs vs. subpleural fibrotic ILA) was performed.

The prevalences of the causes of death and treatment complications were compared between the groups with and without ILAs by Fisher’s exact test.

All statistical analyses were performed using R version 4.0.0 software (R Foundation for Statistical Computing, Vienna, Austria). All P-values were two-sided, and P-values less than 0.05 were considered significant.

## Results

### Demographic characteristics and interobserver agreement

The demographic characteristics of the patients are summarized in Table 1. Of the 478 patients, ILAs were absent in 267 (56%), indeterminate in 125 (26%), and present in 86 (18%). The interobserver agreement for ILA scoring was good (κw = 0.62). ILA patients were classified into three subtypes: non-subpleural (n = 21, 24%), subpleural non-fibrotic (n = 32, 37%), and subpleural fibrotic (n = 33, 38%). Of the 478 patients, 360 patients (75%) underwent surgery, and 206 patients (43%) died within 5 years.

**Table 1:** Patients’ demographic characteristics

	All (n = 478)	Non-ILA (n = 267)	ILA (n = 86)	P values
Age (years)	66.8 ± 8.6	64.1 ± 8.6	71.9 ± 6.3	< 0.001*
Sex				
Women	64 (13%)	40 (15%)	5 (6%)	0.026*
Men	414 (87%)	227 (85%)	81 (94%)	
BMI	21.2 ± 3.3	20.6 ± 3.0	21.6 ± 3.5	0.008*
Smoking history				
Never	97 (21%)	46 (17%)	23 (27%)	0.017*
Former	207 (44%)	116 (44%)	42 (49%)	
Current	168 (36%)	104 (39%)	20 (24%)	
Missing	6	1	1	
Pack-year	32.7 ± 30.6	33.2 ± 30.6	36.2 ± 38.1	0.506
Histology				
SCC	458 (96%)	255 (96%)	83 (97%)	1.000
Adenocarcinoma	20 (4%)	12 (4%)	3 (3%)	
Clinical stage				
I	65 (14%)	40 (15%)	12 (14%)	0.995
II	112 (23%)	57 (21%)	20 (23%)	
III	119 (25%)	66 (25%)	22 (26%)	
IVA	76 (16%)	48 (18%)	15 (17%)	
IVB	106 (22%)	56 (21%)	17 (20%)	
Death in 5 years	206 (43%)	103 (39%)	41 (48%)	0.165
Surgery	360 (75%)	203 (76%)	59 (69%)	0.202
Postoperative complication				
All	158 (44%)	81 (40%)	30 (51%)	0.178
Pulmonary	102 (28%)	50 (25%)	21 (36%)	0.134
% emphysema (%)	0.1 (0.0–0.8)	0.1 (0.0–0.5)	0.3 (0.1–1.2)	0.009*
ILA subtype				
Non-subpleural			21 (24%)	
Subpleural non-fibrotic			32 (37%)	
Subpleural fibrotic			33 (38%)	

Patients indeterminate for ILA were excluded from statistical analysis. Age and BMI were expressed as mean ± standard deviation and compared using *t* test; %emphysema was expressed as median (interquartile range) and compared using Mann–Whitney *U* test; categorical variables were expressed as n (%) and compared by Fisher’s exact test. The percentage of the postoperative complication is calculated in the patients with surgery

*ILA* interstitial lung abnormality, *BMI* body mass index, *SCC* squamous cell carcinoma

*P values < 0.05 was considered statistically significant

Compared with the non-ILA group, the ILA group was older (non-ILA, 64.1 ± 8.6; ILA, 71.9 ± 6.3; P < 0.001), had higher BMI (non-ILA, 20.6 ± 3.0; ILA, 21.6 ± 3.5; P = 0.008), and included more men (non-ILA, 227/267 [85%]; ILA, 81/86 [94%]; P = 0.026). There was a significant difference in smoking status and %emphysema between the two groups (smoking status, P = 0.017; %emphysema, P = 0.009), but no significant difference in pack-years (non-ILA, 33.2 ± 30.6; ILA, 36.2 ± 38.1; P = 0.506). There were no significant differences in cancer stage (P = 0.995) or surgery (P = 0.202).

### Survival

**Fig. 3:**
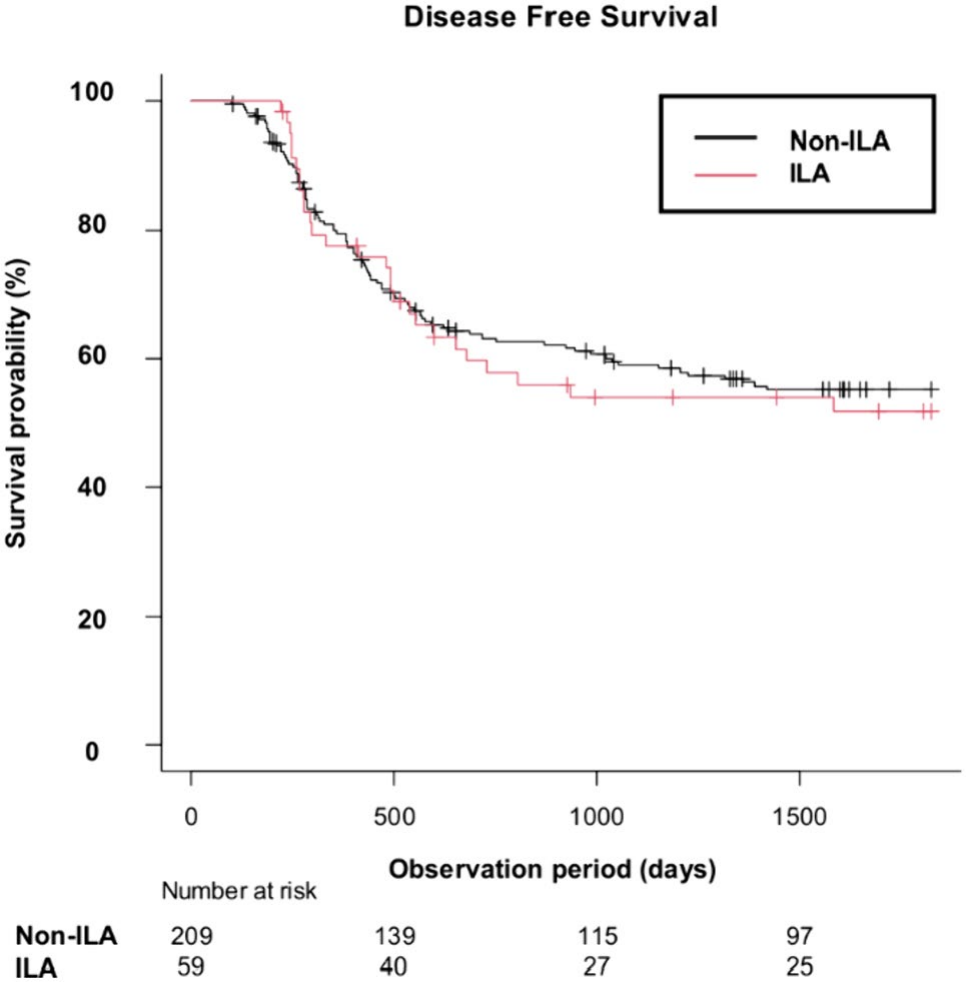
Kaplan–Meier curves for DFS by the presence of ILAs. Non-ILA: DFS events, 89/209 (43%); median DFS (days) NR (95% CI 1318–NR). ILA: DFS events, 27/59 (46%); median DFS (days) NR (95% CI 601–NR). *DFS* disease-free survival, *ILA* interstitial lung abnormality, *NR* not reached, *95% CI* 95% confidence interval

**Fig. 4:**
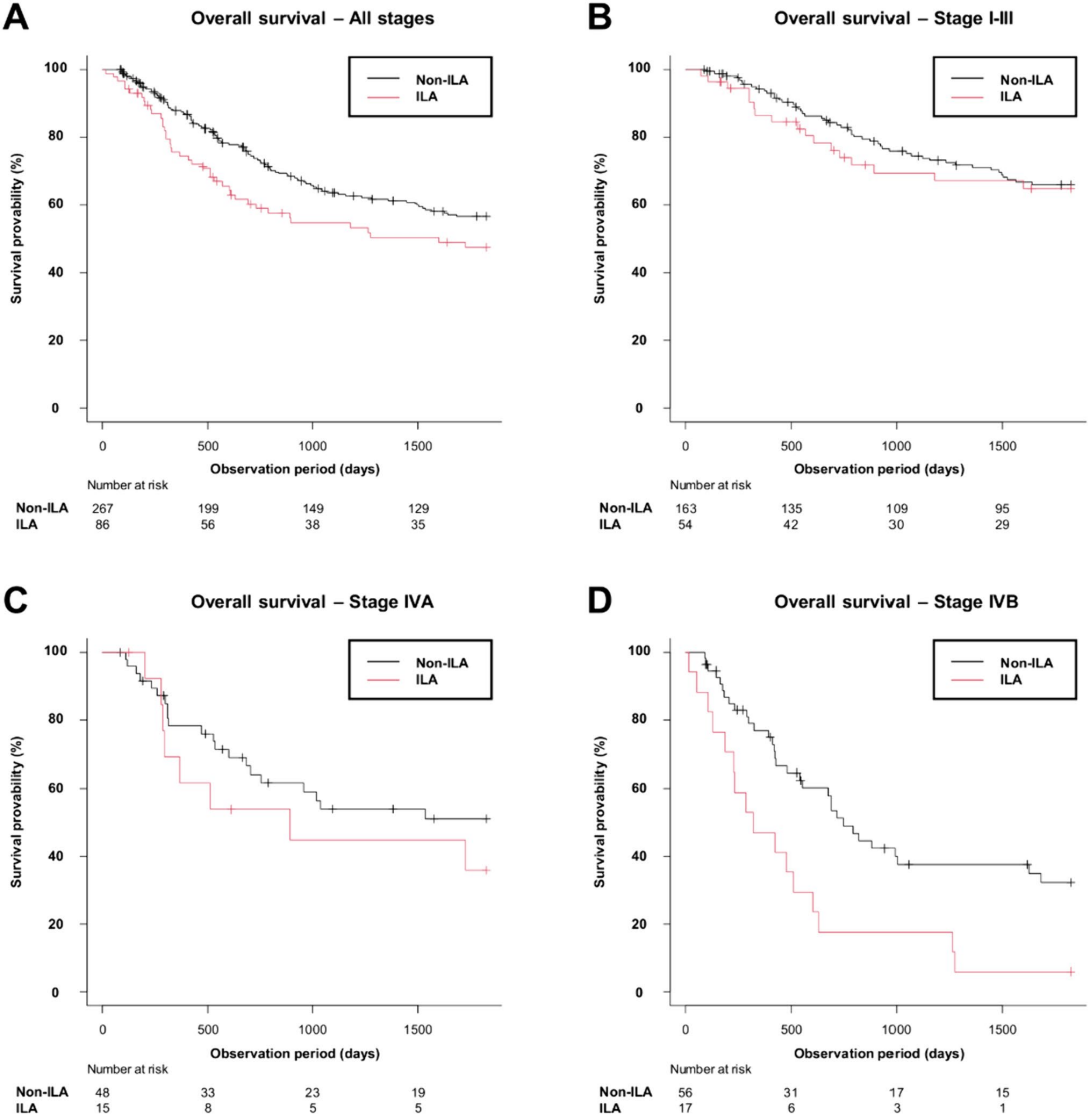
Kaplan–Meier curves for OS by the presence of ILAs. **A** Patients in all stages. Non-ILA: OS events, 103/267 (39%); median OS (days) NR (95% CI 1684–NR). ILA: OS events, 41/86 (48%); median OS (days) 1601 (95% CI 693–NR). **B** Patients with stage I, II and III. Non-ILA: OS events, 50/163 (31%); median OS (days) NR (95% CI NR–NR). ILA: OS events, 17/54 (31%); median OS (days) NR (95% CI 1601–NR). **C** Patients with stage IVA. Non-ILA: OS events, 21/48 (44%); median OS (days) NR (95% CI 706–NR). ILA: OS events, 8/15 (53%); median OS (days) NR (95% CI 287–NR). **D** Patients with stage IVB. Non-ILA: OS events, 32/56 (57%); median OS (days) 752 (95% CI 482–1626). ILA: OS events, 16/17 (94%); median OS (days) 322 (95% CI 131–606). *OS* overall survival, *ILA* interstitial lung abnormality, *NR* not reached, *95% CI* 95% confidence interval

The Kaplan–Meier curves for DFS and OS are shown in Figs. 3 and 4, and the results of the Cox proportional hazards models are summarized in Table 2. In the Cox proportional hazards models, ILA was not a significant factor for worse DFS compared with non-ILA in the univariable analysis (HR = 1.06 [95% CI 0.69–1.63], P = 0.795) and the multivariable analysis (HR = 1.25 [95% CI 0.78–2.01], P = 0.359) adjusting for age, sex, smoking history, clinical stage, and histology. Median follow-up period for DFS was 1196 days.

**Table 2:** HRs of ILAs for worse survival in the Cox proportional hazard models

	Univariable models		Multivariable models	
	HR (95% CI)	P value	HR (95% CI)	P value
Using all stages				
DFS^†^	1.06 (0.69–1.63)	0.795	1.25 (0.78–2.01)	0.359
OS^†^	1.43 (0.99–2.05)	0.054	1.68 (1.10–2.55)	0.016*
Subgroup analysis				
OS in stage I-III^†^	1.14 (0.66–1.97)	0.647	1.19 (0.65–2.19)	0.572
OS in stage IVA^§^	1.49 (0.66–3.38)	0.334	2.31 (0.90–5.94)	0.083
OS in stage IVB^§^	2.48 (1.35–4.56)	0.003*	3.78 (1.67–8.54)	0.001*

ILA was compared with non-ILA

*HR* hazard ratio, *ILA* interstitial lung abnormality, *95% CI* 95% confidence interval, *DFS* disease free survival, *OS* overall survival

^†^Adjusting for age, sex, BMI, smoking history, clinical stage, and histology in the multivariable models

^§^Adjusting for age, sex, BMI, smoking history, and histology in the multivariable models

*A p-value of < 0.05 was considered significant

In terms of OS, though the presence of ILAs was not a significant factor for worse survival in the univariable analysis (HR = 1.43 [95% CI 0.99–2.05], P = 0.054), it was significant in the multivariable analysis (HR = 1.68 [95% CI 1.10–2.55], P = 0.016) adjusting for age, sex, smoking history, clinical stage, and histology. Median follow-up period for OS was 1111 days. In the subgroup multivariable analysis using the patients with stage IVB, the presence of ILAs was a significant factor (HR = 3.78, [95% CI 1.67–8.54]; P = 0.001). On the other hand, in the patients with stage I–III and IVA, the presence of ILAs was not a significant factor for shorter OS (stage I–III: HR = 1.19 [95% CI 0.65–2.19]; P = 0.572; stage IVA: HR = 2.31 [95% CI 0.90–5.94]; P = 0.083).

**Fig. 5:**
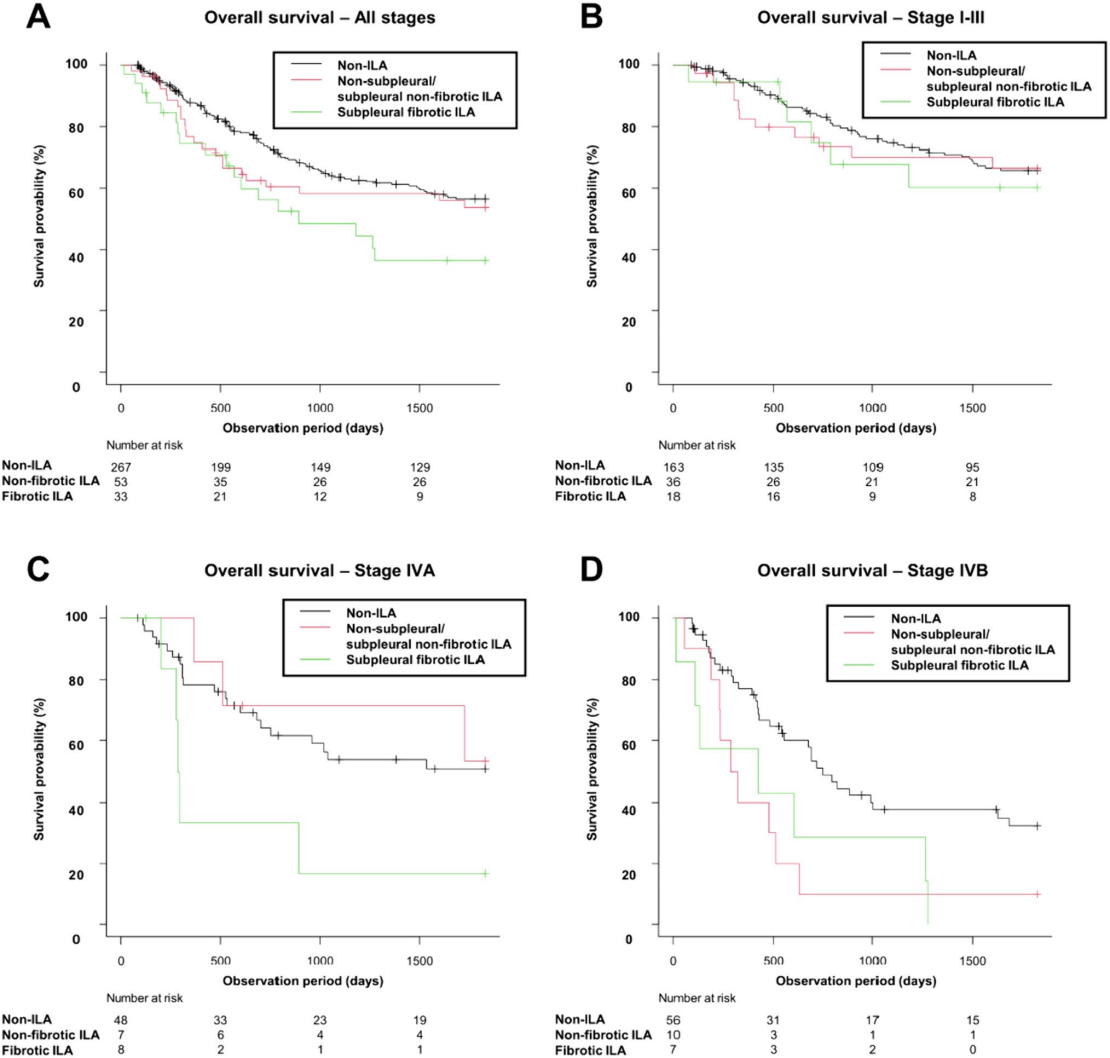
Kaplan–Meier curves for OS split by non-ILAs, non-subpleural and subpleural non-fibrotic ILAs, and subpleural fibrotic ILAs. **A** Patients in all stages. Non-ILA: OS events, 103/267 (39%); median OS (days) NR (95% CI 1684–NR). Non-subpleural and subpleural non-fibrotic ILA: OS events, 23/53 (43%); median OS (days) NR (95% CI 608–NR). Subpleural fibrotic ILA: OS events, 18/33 (55%); median OS (days) 891 (95% CI 532–NR). **B** Patients with stage I, II and III. Non-ILA: OS events, 50/163 (31%); median OS (days) NR (95% CI NR–NR). Non-subpleural and subpleural non-fibrotic ILA: OS events, 11/36 (31%); median OS (days) NR (95% CI 1601–NR). Subpleural fibrotic ILA: OS events, 6/18 (33%); median OS (days) NR (95% CI 693–NR). **C** Patients with stage IVA. Non-ILA: OS events, 21/48 (44%); median OS (days) NR (95% CI 706–NR). Non-subpleural and subpleural non-fibrotic ILA: OS events, 3/7 (43%); median OS (days) NR (95% CI 369–NR). Subpleural fibrotic ILA: OS events, 5/8 (63%); median OS (days) 291 (95% CI 202–NR). **D**: Patients with stage IVB. Non-ILA: OS events, 32/56 (57%); median OS (days) 752 (95% CI 482–1626). Non-subpleural and subpleural non-fibrotic ILA: OS events, 9/10 (90%); median OS (days) 305 (95% CI 54–512). Subpleural fibrotic ILA: OS events, 7/7 (100%); median OS (days) 426 (95% CI 15–1262). *OS* overall survival, *ILA* interstitial lung abnormality, *NR* not reached, *95*%*CI* 95% confidence interval

In the multivariable analysis using ILA subcategories (Table 3 and Fig. 5), subpleural fibrotic ILAs were significantly associated with shorter OS (HR = 2.22 [95% CI 1.25–3.93], P = 0.006), but non-subpleural and subpleural non-fibrotic ILAs were not (HR = 1.44 [95% CI 0.88–2.36], P = 0.146) in the patients with all stages. In the subgroup analysis, subpleural fibrotic ILAs were a significant factor for shorter OS in both the patients with stage IVA and those with stage IVB (stage IVA: HR = 9.41 [95% CI 2.37–37.36], P = 0.001; stage IVB: HR = 4.79 [95% CI 1.35–16.99], P = 0.015) in the multivariable analysis. Non-subpleural and subpleural non-fibrotic ILAs were significantly associated with shorter OS in the patients with stage IVB (HR = 3.54 [95% CI 1.48–8.49], P = 0.005).

**Table 3:** HRs in subcategories of ILA for worse OS in the Cox proportional hazard models

	Univariable models		Multivariable models	
	HR (95% CI)	P value	HR (95% CI)	P value
Non-subpleural and subpleural non-fibrotic ILA				
OS in all stages^†^	1.21 (0.77–1.91)	0.399	1.44 (0.88–2.36)	0.146
OS in stage I-III^†^	1.09 (0.57–2.09)	0.802	1.12 (0.56–2.25)	0.751
OS in stage IVA^§^	0.83 (0.25–2.77)	0.757	1.12 (0.31–4.09)	0.862
OS in stage IVB^§^	2.57 (1.21–5.44)	0.014*	3.54 (1.48–8.49)	0.005*
Subpleural fibrotic ILA				
OS in all stages^†^	1.84 (1.12–3.04)	0.017*	2.22 (1.25–3.93)	0.006*
OS in stage I-III^†^	1.24 (0.53–2.90)	0.615	1.36 (0.55–3.32)	0.504
OS in stage IVA^§^	2.94 (1.09–7.91)	0.032*	9.41 (2.37–37.36)	0.001*
OS in stage IVB^§^	2.39 (1.05–5.43)	0.038*	4.79 (1.35–16.99)	0.015*

*HR* hazard ratio, *ILA* interstitial lung abnormality, *95% CI* 95% confidence interval, *OS* overall survival

^†^Adjusting for age, sex, BMI, smoking history, clinical stage, and histology in the multivariable models

^§^Adjusting for age, sex, BMI, smoking history, and histology in the multivariable models

*A p-value of < 0.05 was considered significant

Similar results were obtained in the multivariable analysis adjusting for age, sex, smoking history, clinical stage, histology, and %emphysema (Supplementary Table A1).

### Causes of death and treatment complications

The causes of death are summarized in Table 4. There was no significant difference in the prevalence of death due to the original lesion of the esophageal cancer (non-ILA, 82/95 [86%]; ILA, 32/39 [82%]; P = 0.596). On the other hand, the ILA group showed more deaths due to pneumonia or respiratory failure (non-ILA, 2/95 [2%]; ILA, 5/39 [13%]; P = 0.022). No patient died due to interstitial lung diseases.

**Table 4:** Summary of causes of death

	Non-ILA (n = 95)	ILA (n = 39)	P value
Original cancer	82 (86%)	32 (82%)	0.596
Pneumonia/respiratory failure	2 (2%)	5 (13%)	0.022*
Aspiration pneumonia	1 (1%)	3 (8%)	
Postoperative pneumonia	1 (1%)		
Respiratory failure secondary to pneumothorax		1(3%)	
Respiratory failure secondary to asphyxia due to sputum		1 (3%)	
Other cancer	8 (8%)	0 (0%)	0.104
Others	4 (4%)	2 (5%)	1.000

Patients with unknown cause of death were excluded (Non-ILA, n = 7; ILA, n = 2). Comparisons were performed using Fisher’s Exact-test

*ILA* interstitial lung abnormality

*P values < 0.05 was considered statistically significant

In terms of postoperative complications, the prevalence tended to be higher in the ILA group than in the non-ILA group, but the difference was not significant (all types of complications: non-ILA, 81/203 [40%]; ILA, 30/59 [51%]; P = 0.178; pulmonary complications: non-ILA, 50/203 [25%]; ILA, 21/59 [36%]; P = 0.134).

Of the patients without surgery, there was no patient with drug-related pneumonitis in the first line therapy or radiation pneumonitis in both the groups with and without ILAs. Of the 478 patients, 18 patients had ICI as a treatment for recurrent lesions (non-ILA, 12/267 [4.5%]; indeterminate for ILA, 2/125 [1.6%]; ILA, 0/86 [0%]) and two non-ILA patients showed pneumonia/pneumonitis. It was not possible to determine whether the pneumonia was immune-related adverse events because of limited available information.

## Discussion

The present results showed a significant association between ILAs and shorter OS in patients with esophageal cancer (HR = 1.68 [95% CI 1.10–2.55], P = 0.016), especially in stage IVB (HR = 3.78, [95% CI 1.67–8.54]; P = 0.001). The prevalence of death due to pneumonia/respiratory failure was higher in the ILA group (non-ILA, 2/95 [2%]; ILA, 5/39 [13%]; P = 0.022). There was no significant difference in postoperative complications between the groups with and without ILAs. Drug-related pneumonitis in the first line therapy and radiation pneumonitis were not observed in the patients without surgery.

Tseng et al. [32] evaluated locally advanced esophageal cancer and reported that there was no association between ILAs and worse mortality. Compared with their study, the present study included metastatic patients (stage IVB). In addition, all patients were Asian, and most patients had SCC in the present study, whereas most patients were White and had adenocarcinoma in the study by Tseng et al. These differences in the cohorts might have influenced the results.

The association between ILA and worse mortality has been reported in various cohorts, including a health screening cohort [35]. These studies suggest that the presence of ILAs affects survival even in individuals without cancer. In the present study, the difference in OS between the groups with and without ILAs was not significant in patients with stage I–IVA, but it was significant in patients with stage IVB. The present results might be caused by not only the general effect of ILA, but also a specific effect in advanced esophageal cancer, because patients with early stage should be influenced more by the general effect of ILA. However, it is unclear why ILAs were associated with a worse prognosis in patients with stage IVB. In the analysis of causes of death including the patients in all stages, the prevalence of death due to pneumonia/respiratory failure tended to be higher in the ILA group than in the non-ILA group. In our speculation, the patients with ILAs might be vulnerable to pneumonia. The damage of pneumonia might be severe in patients with stage IVB and poor general condition. In addition, chemoradiotherapy in patients with stage IVB might increase the prevalence of aspiration pneumonia [36]. However, further investigation is required.

In patients in all stages, subpleural fibrotic ILAs were significantly associated with shorter OS, whereas non-subpleural and subpleural non-fibrotic ILAs were not. Chae et al. [37] investigated patients with ILAs who underwent surgical lung biopsy (n = 45) and reported that subpleural fibrotic ILAs were associated with a higher risk of death than subpleural non-fibrotic ILAs (HR = 9.22 [95% CI 1.23–1180.14]; P = 0.025). Lee et al. [35] investigated an Korean health-screening cohort (n = 2765) and reported that the presence of fibrotic ILAs was a significant factor for worse all-cause mortality compared with non-ILAs (HR = 2.5 [95% CI 1.6–3.8]; P < 0.001), but the presence of non-fibrotic ILAs was not (HR = 1.6 [95% CI 0.7–3.4]; P = 0.23). The present results showed a similar trend to these previous results, and fibrotic ILAs should be considered a significant comorbidity. However, it should be noted that, in the study by Lee et al. non-fibrotic ILAs were associated with a higher risk of mortality related to lung cancer and respiratory causes (HR = 5.3 [95% CI 2.1–13.4]; P < 0.001). In the present study, non-subpleural and subpleural non-fibrotic ILAs were associated with shorter OS in patients with stage IVB. These results suggest that non-subpleural and subpleural non-fibrotic ILAs may also have clinical impact.

Postoperative complications, especially pulmonary infection, are easy to occur and result in critical condition after esophageal cancer surgery because the thoracic wall including intercostal muscles and diaphragm are damaged [38]. In this study, there was no significant difference in postoperative complications. In lung cancers, Im et al. [18] reported that the presence of ILAs was a significant predictor of postoperative pulmonary complication in patients older than 70 years of age with non-small cell lung cancer (NSCLC). They also reported that pneumonectomy and bilobectomy were significant risk factors compared with segmentectomy, wedge resection, and lobectomy. Usual esophageal cancer surgery does not directly invade the lungs, and ILA might have less impact on postoperative pulmonary complications than lung cancer surgery.

Considering that drug-related pneumonitis and radiation pneumonitis were not observed, drug-related pneumonitis and radiation pneumonitis had a small impact on the prognostic difference between the patients with and without ILAs in the stage IVB esophageal cancer group in this study. Nakanishi et al. [21] reported that ground-glass attenuation in ILAs was a significant risk factor for ICI-related pneumonitis (OR = 44.0; P < 0.001) in patients with NSCLC treated with anti-programmed death 1 antibodies. In the present investigation, the most common drugs used for first-line treatment in the patients without surgery were fluorouracil, cisplatin, and docetaxel. Few patients had ICI therapy in this study because the study period predated the general use of ICI for esophageal cancer. In recent practice, ICI plus chemotherapy can be used in first-line treatment for advanced esophageal cancer [39, 40]. The difference in the treatment strategy may result in the different influence of ILA on the prognosis of esophageal cancer.

The present study has several limitations. First, this was a retrospective, single-center study. Second, the evaluation of ILAs was based only on visual interpretation by radiologists. Visual assessment of ILAs has been widely performed in other studies [3–9], and interobserver agreement was good in the present study, but a quantitative approach might be beneficial for reproducibility [41]. Third, images in 17 patients were not thin-section CT (slice thickness < 1.5 mm), which might affect the ILA scoring. Forth, the Asian ethnicity and the heavy male predominance of the patients included in this study might have impacted the results. Finally, there was limited sample size in the subgroup analysis of ILA subcategories and the analysis for cause of death. Especially, the observed difference in the prevalence of death due to pneumonia or respiratory failure between the ILA and non-ILA groups might be affected by confounders such as age.

In conclusion, ILAs were significantly associated with shorter OS in patients with esophageal cancer, especially in patients with stage IVB disease. ILAs may be an important prognostic factor in patients with esophageal cancer, and the present results suggest that patients with ILAs may need careful follow-up.

### Supplementary Information

Below is the link to the electronic supplementary material.

Supplementary file1 (DOCX 29 kb)

## Data Availability

The data that support the findings of this study are not openly available due to reasons of sensitivity and are available from the corresponding author upon reasonable reque st. Data are located in controlled access data storage at Osaka University.
