# Supplementary material for: Association between interstitial lung abnormality and mortality in patients with esophageal cancer
Source: Jpn J Radiol. 2024 Apr 25;42(8):841–51. doi: 10.1007/s11604-024-01563-x (PMC11286667; doi:10.1007/s11604-024-01563-x)
Supplement: Supplementary file 1 — Supplementary file1 (DOCX 29 kb) [file 11604_2024_1563_MOESM1_ESM.docx]

**Supplementary document**

**Materials and Methods**

**- Study population (Patient selection)**

Of the 659 patients identified by the search, 455 patients had CTs with the protocol for baseline evaluation of esophageal cancer. Out of the remaining 204 patients, 23 patients had available CTs before treatment (our hospital, n=8; other hospital n=15). Therefore, a total of 478 patients (455 plus 23) were included in this study.

Excluded tumors of histological types other than adenocarcinoma and SCC were as follows; basaloid-squamous cell carcinoma, n=2; carcinosarcoma, n=4; melanoma, n=2; neuroendocrine tumor, n=5; undiagnosed tumor, n=3.

**- CT acquisition and image reconstruction**

For the 455 patients with CT scans performed using the scan protocol for baseline evaluation of esophageal cancer, contrast-enhanced (CE) CT was performed at full inspiration in the supine position. A total of 95 mL of contrast medium was administered intravenously at a rate of 2 mL/s, and CT images were acquired 50 seconds after the start of contrast administration. Images were reconstructed with slice thickness of 0.5 mm or 0.625 mm and the soft tissue reconstruction kernel. Of eight additional patients at our hospital, four patients underwent only non-CE CT, and four patients had only 5-mm-thick images. The CT scanners used were Aquilion/Aquilion One/Aquilion Prime (n=7/203/1, Toshiba/Canon Medical Systems, Otawara, Japan), LightSpeed VCT/Discovery CT 750HD (n=54/197, GE Medical Systems, Milwaukee, WI), and Sensation16 (n=1, Siemens Medical Solutions, Forchheim, Germany). The other parameters for scanning and reconstruction were as follows: tube voltage, 120 kVp; tube current, automatic tube current modulation; matrix size, 512 x 512; field of view, 345 mm.

The CT scans in 15 patients performed at the other hospitals included 8 CE CTs and 7 non-CE CTs.

In the evaluation of ILA, thin-section images with thickness of 0.5 or 0.625 mm were used for evaluation for the CTs performed with our baseline protocol (n=455). For the other CT examinations, images with the thinnest slice thickness (0.5/0.625 mm, n=4; 1.25 mm, n=2; 3.75 mm, n=1; 5 mm, n=13; 7 mm, n=3) were evaluated. In the interpretation process, a commercially available viewer (SYNAPSE SAI viewer, FUJIFILM Corporation, Tokyo, Japan) was used, and a sharpness filter was applied to the images reconstructed using the soft tissue kernel.

**Emphysema quantification**

Emphysema quantification on CT was obtained in the groups without and with ILA. Non-contrast-enhanced CT images with 5-mm slice thickness and a smooth kernel were used for the quantification [1]. The severity of emphysema was evaluated by using the percentage lung volume occupied by low-attenuation areas (voxels with attenuation of −950 HU or less; %emphysema). We used a software program to perform the quantification (3DSlicer; <http://www.slicer.org>) [2].

To validate the quantification, we calculated Pearson's correlation coefficients for the %emphysema and pack-years of cigarette smoking. In addition, the correlation with the forced expiratory flow in 1 second (FEV1) and the ratio of FEV1 to forced vital capacity (FEV1/FVC) were also calculated in the patients with pulmonary function test results.

**- Causes of death and treatment complication**

Pulmonary postoperative complications included pneumonia, respiratory failure, acute respiratory distress syndrome, significant atelectasis requiring bronchoscopy or reintubation, bronchopleural fistula/empyema, prolonged air leakage lasting for more than 5 days, and pneumothorax [3].

Symptomatic drug-related pneumonitis and/or radiation pneumonitis (Common Terminology Criteria for Adverse Events grade 2 or more) were recognized and diagnosed according to the following criteria: 1) newly emerged lesion, 2) ground-glass attenuation or infiltrative shadow on CT, 3) no indication of lung infection including purulent sputum, improvement by antibiotics, and positive results for sputum and/or blood cultures. Abnormal shadow with pulmonary invasion of cancer, a relevant bronchial obstruction, or apparent heart failure were excluded [4]. Drug-related pneumonitis was investigated in first-line therapy.

**Results**

Emphysema quantification was obtained in 341 patients without and with ILA. 12 patients were excluded from the emphysema analysis because they did not have non-contrast-enhanced CT images before treatment. Among the 341 patients, 282 patients had pulmonary function test results.

The correlation coefficients of %emphysema were 0.145 (P=0.012) for pack-year, -0.391 (P<0.001) for FEV1 percent predicted, and -0.386 (P<0.001) for FEV1/FVC.

**References**

1. Gierada DS, Bierhals AJ, Choong CK, Bartel ST, Ritter JH, Das NA, et al. Effects of CT Section Thickness and Reconstruction Kernel on Emphysema Quantification: Relationship to the Magnitude of the CT Emphysema Index. Acad Radiol. 2010;17:146.

2. Lynch DA, Moore CM, Wilson C, Nevrekar D, Jennermann T, Humphries SM, et al. CT-based Visual Classification of Emphysema: Association with Mortality in the COPDGene Study. Radiology. 2018;288:859–66.

3. Im Y, Park HY, Shin S, Shin SH, Lee H, Ahn JH, et al. Prevalence of and risk factors for pulmonary complications after curative resection in otherwise healthy elderly patients with early stage lung cancer. Respiratory Research. 2019;20:1–9.

4. Shibaki R, Ozawa Y, Noguchi S, Murakami Y, Takase E, Azuma Y, et al. Impact of pre-existing interstitial lung abnormal shadow on lung injury development and severity in patients of non-small cell lung cancer treated with osimertinib. Cancer Medicine. 2022;11:3743–50.

Table A1 HRs in the multivariable Cox proportional hazard models adjusting for %emphysema.

|  | Multivariable models | |
| --- | --- | --- |
| Using all stages | HR (95% CI) | P value |
| DFS† | 1.23 (0.76-2.02) | 0.396 |
| OS† | 1.69 (1.10-2.59) | 0.016* |
|  |  |  |
| Subgroup analysis | HR (95% CI) | P value |
| OS in stage I-III† | 1.19 (0.64-2.21) | 0.592 |
| OS in stage IVA§ | 2.50 (0.96-6.52) | 0.061 |
| OS in stage IVB§ | 3.78 (1.67-8.54) | 0.001* |
|  |  |  |
| Non-fibrotic ILA | HR (95% CI) | P value |
| OS in all stages† | 1.45 (0.87-2.39) | 0.150 |
| OS in stage I-III† | 1.09 (0.53-2.25) | 0.811 |
| OS in stage IVA§ | 1.20 (0.32-4.43) | 0.787 |
| OS in stage IVB§ | 3.54 (1.48-8.49) | 0.005* |
|  |  |  |
| Fibrotic ILA | HR (95% CI) | P value |
| OS in all stages† | 2.25 (1.26-4.00) | 0.006* |
| OS in stage I-III† | 1.40 (0.56-3.49) | 0.467 |
| OS in stage IVA§ | 13.17 (3.02-57.44) | 0.001* |
| OS in stage IVB§ | 4.80 (1.35-17.06) | 0.015* |
| †Adjusting for age, sex, BMI, smoking history, clinical stage, histology, and %emphysema in the multivariable models.  §Adjusting for age, sex, BMI, smoking history, histology, and %emphysema in the multivariable models.  *A p-value of <0.05 was considered significant.  HR, hazard ratio; ILA, interstitial lung abnormality; 95%CI, 95% confidence interval; OS, overall survival. | | |
